# Filling Teeth and the Philosophy Thereof

**Published:** 1883-09

**Authors:** J. Hayhurst

**Affiliations:** Lambertville, N. J.


### FILLING TEETH AND THE PHILOSOPHY THEREOF.

BY DR. J. HAYHURST, LAMBERTVILLE, N. J.

In considering the subject which we have chosen to-day, it will be necessary to allude to that which causes so much labor, and to counteract which calls forth the most skillful manipulations of the most scientific and adroit members of our profession. I mean

DENTAL CARIES.

It is not my purpose just now to enter into an elaborate and detailed investigation of the causes of this disease, or into a critical analysis of the agents which work so great a harm to the human family, but merely to state in a general way what it is, and so far to describe it as to enable us to know the nature of the enemy with which we have to contend.

Dental caries is a breaking down of the tooth-body, occasioned by the external application of some agent inimical to the health of the tooth, or it is an inherent vice growing out of the pathological condition of the patient, operating from within outward—the former from the outside inward—devitalizing and destroying the tooth structure.

As to whether or not this agent is acid, alkaline, mechanical or parasitical, it is not my intention to inquire.

If any or all these agents are at work, then it is plain that we can, by properly routing them out of their lodgment and preventing their re-entrance, stop their devastation. But if we fail to close the door after the expulsion of the bad tenant, then it is well described in holy writ: "After the unclean spirit was cast out he says I will return to whence I came. And when he cometh he findeth it swept and garnished. Then he goeth and taketh with him seven other spirits more wicked than himself, and they enter in and dwell there, and the last state of that man is worse than the first."

So it is with the tooth. In our endeavor to repair and preserve, we take out all of the diseased matter, and leave the sound walls of the cavity to receive the filling, if properly placed, and thus prevent further destruction of healthy substance. Thus can we not see that if we do not shut the door close against the entrance of the evil, the work of destruction will go on with increased force, and thus the last stages of that tooth will be far worse than the first.

If our operation is imperfectly done, and we do not make a tight union between the substance used as a filling and the cavity, thus permitting the corroding agent to enter between the filling and the newly exposed bony structure, the plug will assist in holding the injurious matter up to its work.

It can make no difference in the result as to whether the material out of which we propose to make the filling is hard or soft, spongy or dense, plastic or solid, if we leave the margin between the filling and the sides of the cavity so that the corrosive fluid can find a lodgment there. The trouble lies not in the material employed, but in the manner of its introduction into its place. It is an almost universally admitted fact that the fillings that most nearly accomplish the design of the manipulator are those of gold foil, properly made and introduced, and here is a point to which I wish to draw your attention. Let us examine some of the materials used, and find out if possible what are the objections to them. Gold, fine gold, is incorruptible, and for aught I know sponge gold or plastic gold is as fine as any other. It claims to be pure, yet from most operators comes up the cry, "I can not make good work with it—let me take as much care as possible, make it as dense as I can, put as fine a polish as I please, it goes out from under my hand to all appearances a perfect specimen of workmanship, but in a short time the patient comes back, and there is a dark appearance visible between the filling and the enamel. How comes it? What is the cause ?"

The mechanical arrangement of the condensed fillings of plastic gold and amalgam are the same, the body of the filling becoming very dense. It is of a homogeneous structure, and the laws of expansion and contraction are the same in similarly constructed bodies.

What is the explanation of the defect in the plastic gold filling ? The gold has retained its integrity; no change there. But there has been sufficient contraction to permit the fluids of the mouth to interpose between the gold and the bone, and hence the mischief.

If we could have the same chance for a nice observation of an amalgam filling, one as free from extraneous matter as the gold, we should find the same cause producing the same effect.

The failure to make a perfect filling is in the mechanical composition of the plug.

In the former portion of this essay I alluded to a gold filling made from gold foil. This kind of a filling is universally admitted to be the best that can be made.

What is its structure? And why should it claim the pre-eminence it has acquired ? It is not, strictly speaking, a homogeneous mass, but is formed of layers or convolutions. When a mass thus formed expands or contracts, it does so in the direction of the lamina or shreds of which it is composed, and this expansion is not a rigid one, but one that will conform in a greater or less degree to the cavity which contains it.

Thus we have a filling that will adjust itself to its surroundings, while the solid mass expands against the walls that confine it, and pushes itself away from the tooth substance, while the laminated mass, not being so rigid, does not act with such force, either against the walls of the cavity or upon itself, as to make a well-defined space between the filling and the tooth-bone. If the theory I have endeavored to establish is correct, then we have done all we can when we have removed the obnoxious substance and replaced it with a pure and clean material, so that the enemy cannot enter to destroy and lay waste the fair heritage with which every healthy being is endowed.

I do not know whether any one has ever claimed that there was any curative property in a filling, but we are often asked if, when the tooth is properly filled, it will ever decay again. We do not promise to make the tooth better than nature had made it, but we propose to repair all damages made by disease, or at least to put it in the best condition possible to resist the progress of decay. But this does not improve the quality of the material of which the tooth is composed.

There is a class of materials of a plastic character that is extensively used amongst us, but as most of them are of a temporary nature I leave the subject with but this slight notice of them. Much more might be said on this interesting subject, but as it is impossible to exhaust it I leave it in the hope that this presentation may tend to bring out something from others.

